# Health Data Quality Skill Gaps and Training Needs Among European Health Data Stakeholders: Cross-Sectional Survey

**DOI:** 10.2196/86878

**Published:** 2026-07-06

**Authors:** Jens Declerck, Niina Eklund, Carlos Sáez, Peter Rijnbeek, Enrique Bernal-Delgado, Erik van Mulligen, Dipak Kalra

**Affiliations:** 1 The European Institute for Innovation through Health Data Ghent Belgium; 2 Ghent University Ghent, Flanders Belgium; 3 BBMRI-ERIC Graz Austria; 4 Universitat Politècnica de València (UPV) Instituto Universitario de Tecnologías de la Información y Comunicaciones (ITACA) Biomedical Data Science Lab Valencia Spain; 5 Erasmus University Medical Center Rotterdam The Netherlands; 6 Data Science for Health Services and Policy Research Institute for Health Sciences (IACS) Zaragoza Spain; 7 Erasmus University Medical Center Department of Medical Informatics Rotterdam The Netherlands

**Keywords:** European Health Data Space, health data, data quality, data secondary use, data holders, data users, health data access bodies, capacity building, QUANTUM project, data quality labeling

## Abstract

**Background:**

The secondary use of health data is accelerating across Europe driven by growing demand for data-enabled research, innovation, and policymaking. The European Health Data Space (EHDS) establishes a regulatory framework to support this ecosystem, including Article 78, which mandates a data quality and utility labeling mechanism for datasets intended for reuse. Implementing this framework requires that data holders, data users, and health data access bodies possess sufficient skills, training, and organizational capacity to assess, document, and communicate data quality. However, little empirical evidence exists on whether European health data stakeholders currently possess these capabilities or how their needs differ across the 3 EHDS-defined roles.

**Objective:**

This study aimed to identify current skill gaps, training needs, and organizational readiness related to health data quality among European health data stakeholders within the context of the EHDS.

**Methods:**

A cross-sectional online survey was conducted between March 2024 and April 2024 using convenience sampling through the QUANTUM (Quality, Utility, and Maturity Measured; Developing a Data Quality and Utility Label for the European Health Data Space) consortium network, professional mailing lists, and health data communities. The survey targeted individuals involved in the secondary use of health data who identified as data holders, data users, or health data access bodies. The survey assessed 5 domains: stakeholder roles and data interaction, individual skills and experience, perceived challenges and skill gaps, organizational support and tools, and learning needs and preferences. Overall, 64 responses were collected from participants representing 44 institutions across 18 European countries.

**Results:**

Overall, 82.8% (53/64; 95% CI 71.8%-90.1%) of respondents interacted with health data at least weekly, and 84.4% (54/64; 95% CI 73.6%-91.3%) rated data quality as moderately to absolutely critical for their work. Despite this, 87.5% (56/64; 95% CI 77.2%-93.8%) reported that poor data quality limited their effectiveness, with missing or inconsistent data identified as the most prevalent challenge. While 79.7% (51/64; 95% CI 67.4%-88.3%) reported prior experience with data quality tasks, key skill gaps were identified in applying data quality metrics, auditing and reporting, and metadata management. At the organizational level, only 15.6% (10/64; 95% CI 8.7%-26.6%) reported clearly defined data quality roles, and 68.8% (44/64; 95% CI 56.6%-78.8%) lacked a dedicated data quality manager or team.

**Conclusions:**

This study provides an empirical assessment of data quality skills and organizational readiness across the 3 EHDS-defined stakeholder groups. The findings highlight that practical experience alone does not ensure data quality competence and that structural deficits, particularly unclear roles and limited governance, constrain effective data quality management. The results offer a role-specific, evidence-based road map for capacity-building efforts essential to the successful implementation of the EHDS Article 78 data quality and utility labeling framework. This evidence underscores the urgent need for coordinated capacity building to ensure successful EHDS data quality implementation.

## Introduction

Across Europe, there is growing momentum around the secondary use of health data for research [1], innovation [2], and policymaking [3]. Health data are increasingly being reused to develop artificial intelligence models [4], inform evidence-based policy [5], and improve population health outcomes [6]. However, alongside these opportunities comes a growing awareness of the challenges related to the quality of health data [7,8]. As health datasets originally collected for clinical, primary research, or administrative intentions are repurposed for secondary use, determining whether those data are truly fit for use becomes a critical concern—one that, if left unaddressed, could reduce trust in data sharing infrastructures. Ensuring this fitness for purpose often depends on data provenance [9] and clear records of a dataset’s origin, context, and transformations, which support accurate quality assessment and reduce the risk of misinterpretation.

The challenge related to health data quality is particularly relevant within the emerging European Health Data Space (EHDS) [10], where health data are pooled from a wide range of sources (eg, registries and health care) and managed by key stakeholder groups defined in the regulation (eg, data holders, data users, and health data access bodies). The variability in how these data are captured, curated, and governed adds complexity to ensuring their quality, interoperability, interpretability, and utility across use cases. Recognizing this, the EHDS regulation, which came into force on March 26, 2025 [11], establishes a common framework for the European Union for the access, control, and cross-border sharing of personal health data. While Chapter 2 of the regulation addresses the primary use of data for care, Chapter 4 outlines the requirements for secondary use.

A central component of this secondary use framework is Article 78 [12], which mandates the creation of a data quality and utility label for datasets intended for reuse. This label is meant to increase transparency; help users assess whether a dataset meets their specific needs; and, ultimately, promote trust in the health data ecosystem. To support the implementation of these requirements, the European Commission has funded the QUANTUM (Quality, Utility, and Maturity Measured; Developing a Data Quality and Utility Label for the European Health Data Space) project (HORIZON-HLTH-2023-TOOL-05, project 101137057) [13]. One of QUANTUM’s objectives is to develop a labeling framework and technical toolkit for assessing and communicating the quality, utility, and maturity of datasets across the EHDS. However, a key challenge lies in the readiness of stakeholders (eg, data holders, health data access bodies, and data users), as data holders are responsible for labeling the datasets they manage, whereas all 3 groups must be able to interpret and apply these labels effectively. While the regulatory expectations are clear, there is limited insight into how prepared these stakeholders are to meet these requirements [14] or how their capacity-building needs might differ across the 3 EHDS roles.

This gap is particularly present in the context of the EHDS. A 2025 European Federation of Pharmaceutical Industries and Associations position paper explicitly called for targeted stakeholder engagement and capacity building as a prerequisite for successful EHDS implementation [15], and a 2023 Sitra report on advancing data sharing in Europe similarly identified capacity gaps as a structural barrier to realizing the benefits of health data reuse [14]. The EHDS regulation places concrete obligations on data holders to produce quality labels under Article 78 and on data users and health data access bodies to interpret and act on them. However, there is little evidence on whether these groups are currently equipped to meet these obligations or how their capacity-building needs might differ across the 3 EHDS roles. Without investing in these foundational capabilities, there is a risk that labeling efforts may become a box-ticking exercise rather than a tool that genuinely improves the reusability and reliability of health data. To address this gap, this study explored the following research question: what are the current skill gaps and training needs among European health data stakeholders in assessing and managing data quality? By identifying practical challenges and capacity gaps, particularly from the perspective of those working with health data quality, this paper aims to inform the design of educational strategies under the EHDS.

## Methods

### Study Design

This study used a cross-sectional exploratory survey design to investigate current skill gaps and training needs among European health data stakeholders in managing data quality and data quality labeling. The work was conducted under the QUANTUM project, which supports the development of data quality and utility labeling mechanisms within the EHDS. QUANTUM brings together stakeholders from across Europe to improve the interoperability, reliability, and secondary use of health data for research, policy, and innovation.

### Inclusion and Exclusion Criteria

Eligible participants were actively involved in the secondary use of health data in a professional capacity and identified with at least 1 of the 3 EHDS-defined stakeholder roles: data holder, data user, or health data access body. Participants were required to be affiliated with a European institution or organization operating within the European health data landscape. No language restrictions were applied. Individuals who did not interact with health data in a professional context were excluded. Participation was limited to one response per individual. No restrictions were placed on country of residence within Europe, institutional size, or sector.

### Survey Development

The survey was developed by a multidisciplinary team of data quality experts led by the European Institute for Innovation Through Health Data (i~HD); Biobanking and Biomolecular Resources Research Infrastructure–European Research Infrastructure Consortium; and Sciensano, Belgium’s national institute for health, drawing on the EHDS stakeholder typology and the QUANTUM project’s objectives. The instrument was initially drafted and reviewed internally by the expert team and then piloted informally at the Ghent i~HD conference in 2023 with a small group of health data professionals. Feedback from this pilot was used to refine item wording and improve clarity. As this was a purpose-built exploratory instrument rather than a formally validated scale, full psychometric validation (including factor analysis or criterion validity testing) was not conducted. This is acknowledged as a limitation. The survey used a combination of item types: Likert-type rating scales (including a 5-point self-assessment scale from 1=“no understanding” to 5=“expert understanding”), multiple-choice single- and multi-selection items, and open-text fields.

The survey was structured to collect both quantitative and qualitative data across 5 key domains. The first domain focused on stakeholder roles and data interaction, aiming to identify how respondents engaged with health data and what organizational roles they occupied. The second domain assessed individual skills and experience, capturing respondents’ knowledge of data quality concepts, their prior training, and their personal involvement in practical data quality assessments. The third domain explored perceived challenges and skill gaps, seeking insights into common data quality issues encountered and specific areas where respondents felt underprepared. The fourth domain examined organizational support and tools, evaluating the presence of data governance structures, the use of data quality tools, and overall perceptions of organizational readiness for managing health data quality. Finally, the fifth domain addressed learning needs and preferences, gathering information on preferred training formats, time commitment, and the features that respondents valued most in educational programs.

In alignment with the stakeholder typology defined by the EHDS legislative framework, the survey explicitly asked participants to identify their roles as data holders, data users, or data access bodies. Respondents were allowed to select multiple roles, reflecting the reality that many organizations operate across different capacities within the health data ecosystem. The prevalence of multi-role respondents was not treated as a sampling limitation but as a substantive finding in itself: it reflects the reality that, in the European health data ecosystem, stakeholder roles are transactional rather than fixed.

### Sampling Procedures

Participants were recruited using convenience sampling via 3 primary channels. Stakeholders were invited to participate in the survey through targeted outreach conducted via the QUANTUM consortium network, professional mailing lists, and relevant health data communities. The QUANTUM consortium comprises approximately 20 partner organizations across 12 European countries. The survey link was circulated via direct email to consortium contacts and posted in community forums and newsletters. The survey targeted a broad spectrum of individuals involved in the secondary use of health data. The survey was conducted online using LimeSurvey (LimeSurvey GmbH), a secure and General Data Protection Regulation–compliant platform, from March 2024 to April 2024. The exact size of the distribution list was not systematically tracked. Therefore, a precise response rate cannot be calculated, which is acknowledged as a limitation of the study design.

### Data Analysis

No formal a priori power calculation was conducted, consistent with the exploratory and descriptive nature of this study. This study aimed to collect as many responses as feasible within the available network and project timeline, yielding a total of 64 responses. This sample size is not intended to support confirmatory inferential testing. Precision of estimates is communicated through 95% Wilson score CIs reported for all proportions. Item-level missing data varied across items and are reported alongside each result. Given the convenience sampling approach, the small total sample, and the nonprobability nature of recruitment, inferential statistical testing was not performed.

To assess the nature of item-level missing data, the Little missing completely at random (MCAR) test was applied to the 12 core variables (N=64; 768 total cells) [16]. Multiple imputation was subsequently performed using iterative chained equations to verify the robustness of complete-case estimates [17].

### Ethical Considerations

This study was conducted as an anonymous, voluntary online survey targeting professionals in a nonsensitive context. No clinical or sensitive personal health data were collected from participants. Survey responses were collected and stored on a General Data Protection Regulation–compliant platform hosted by the i~HD. Data access was restricted to members of the research team. Participation was entirely voluntary. No financial or material compensation was provided to respondents. Participants were surveyed only in their professional capacity about their skills and training needs. The survey was carried out within the QUANTUM project, which underwent the mandatory Horizon Europe ethics appraisal as part of its grant agreement [18,19]. Within the project, an anonymous noninterventional survey of professionals collecting no personal health data did not require approval by a research ethics committee [18,19]. Such approval was therefore not sought for this study. No images or materials that could enable identification of individual participants are included in the manuscript or supplementary material.

## Results

### Overview

A total of 64 responses were collected from stakeholders across 18 European countries, representing 44 different organizations, with the highest participation from Croatia followed by Belgium and Italy. Respondents represented a range of roles within the health data ecosystem. The survey attracted 15 unique data holders who exclusively managed data without overlapping into use or access body roles, 4 data users who primarily leveraged data for various applications, and 5 dedicated data access bodies responsible for facilitating data availability. In addition, 13 institutions reported multiple roles, engaging in both holding and use of data or combining all 3 functions. This multi-functionality reflects the integrated and interdependent nature of the European health data ecosystem.

The level of missing data was low (6/768, 0.8% of cells), confined to 4 items: impact of poor data quality (1/64, 1.6%), self-assessed understanding (1/64, 1.6%), ability to resolve quality issues (1/64, 1.6%), and clarity of data quality roles (3/64, 4.7%). The Little MCAR test yielded *χ*^2^_11_=19.9 (*P*=.047), suggesting a missing at random rather than completely random pattern. Multiple imputation with 20 imputed datasets confirmed the robustness of complete-case estimates, with differences of at most 1.6 percentage points and no changes in interpretation for any findings.

In total, 6.3% (4/64) of the respondents did not identify with any of these 3 stakeholder categories and described themselves as patient representatives or health data consultants. For role-specific analysis, responses were grouped according to each selected role. Therefore, individuals indicating multiple roles may appear in more than one category.

### Stakeholder Roles and Interaction With Health Data

The survey revealed that health data played a central role in the daily work of most respondents. A total of 82.8% (53/64; 95% CI 71.8%-90.1%) reported interacting with health data at least weekly, and more than half (34/64, 53.1%; 95% CI 44.1%-67.7%) did so daily, highlighting its relevance across research, policy, and clinical contexts. Data quality was also seen as essential to effective decision-making. A strong majority rated it as moderately to absolutely critical (54/64, 84.4%; 95% CI 73.6%-91.3%), with nearly 1 in 5 (12/64, 18.8%; 95% CI 11.1%-30%) saying that they could not make decisions without ensuring the quality of the data first.

**Figure 1:**
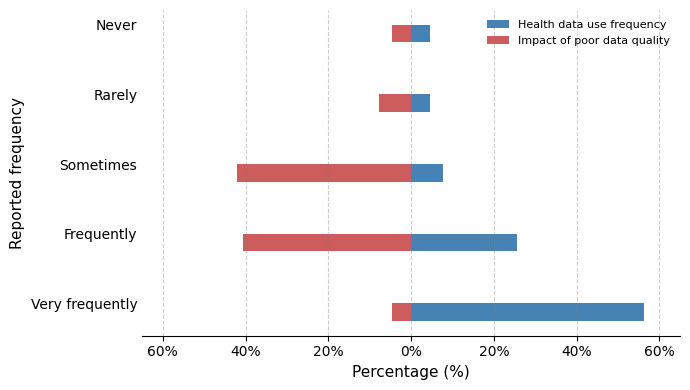
Frequency of health data use (red) and perceived impact of poor data quality (blue) across respondents.

Despite this, an alarming rate of respondents (57/64, 89.1%; 95% CI 79.1%-94.6%) reported that poor data quality limited their effectiveness (Figure 1). For many, this was a common challenge: 40.6% (26/64; 95% CI 29.5%-52.8%) faced quality issues frequently, and a small but important proportion (3/64, 4.7%; 95% CI 1.6%-13%) felt consistently held back. Common problems included missing or inconsistent data, delays in availability, and a lack of data standardization and metadata.

Differences across stakeholder roles were evident. Health data access bodies reported the highest frequency of daily data use and were also most likely to say that poor data quality severely limited their work. In contrast, data holders often found these issues more manageable, whereas data users reported operational impacts such as those of health data access bodies. These variations reflect the differing responsibilities and dependencies that each group has within the health data ecosystem.

### Individual Skills and Experience With Data Quality

Respondents reported mixed levels of understanding when it came to data quality. On a 5-point self-assessment scale, the most common rating was 3 (21/64, 32.8%; 95% CI 22.4%-45.1%), indicating a moderate grasp of the topic. In total, 25% (16/64; 95% CI 16.3%-37.4%) rated themselves at a high level (4 or 5), whereas a smaller group (6/64, 9.4%; 95% CI 4.4%-19%) admitted to having limited understanding (rating of 2). No respondents selected 1 (“no understanding”). Despite these differences, hands-on experience was widespread. A total of 79.7% (51/64; 95% CI 67.4%-88.3%) of respondents had previously assessed the quality of a health dataset, suggesting frequent practical engagement across the community.

Participants gained their knowledge from a variety of sources. The most common were organizational guidelines, formal education such as a master’s degree, and in-house training. Others mentioned external courses and informal learning such as peer exchange or on-the-job experience. However, even with this experience, challenges remained. While 87.5% (56/64; 95% CI 77.2%-93.8%) had encountered data quality errors ranging from missing data to inconsistent formats and poor documentation, only 37.5% (24/64; 95% CI 26.8%-49.6%; Multimedia Appendix 1) said that they often resolved them effectively. Most (29/64, 45.3%; 95% CI 33.8%-57.3%) managed issues only sometimes, and 10.9% (7/64; 95% CI 5.4%-20.9%) struggled to resolve them at all.

Here, again, the difference between the stakeholders was evident. Data users and health data access bodies were more likely to rate their knowledge as high and report success in addressing issues, likely reflecting their deeper involvement in data analysis and processing. Data holders showed more variation, possibly due to the diversity of roles and functions within that group.

### Organizational or Personal Challenges and Skill Gaps

A total of 87.5% (56/64; 95% CI 77.2%-93.8%) of the respondents reported encountering data quality issues in their work, most commonly involving missing, inaccurate, or duplicated data. These problems often stemmed from unstructured inputs, poor annotations, and a lack of validation during data collection. Delayed data availability and insufficient metadata further weakened data users’ trust in the usability of the data.

Beyond technical flaws, respondents highlighted broader structural challenges affecting the quality and consistency of data management. The absence of standardized practices, standard operating procedures, and terminologies, especially across organizations, was seen as a major barrier to data integration and quality control. Documentation gaps, low awareness of data quality principles, and outdated manual processes also featured prominently. Table 1 shows the gaps identified by the respondents.

**Table 1:** Skill gaps identified by the respondents (N=64).

Skill	Respondents, n (%)
Developing and applying data quality metrics	10 (15.6)
Data quality auditing and reporting	8 (12.5)
Metadata management and documentation	7 (10.9)
Training in relevant data quality standards	6 (9.3)
Use of data quality improvement software	6 (9.3)
Advanced data analysis and interpretation	5 (7.8)
Implementation of data governance policies	5 (7.8)
Skills for communicating data quality issues to stakeholders	4 (6.3)
Data integration and consolidation methods	4 (6.3)
Data cleaning techniques	4 (6.3)
Knowledge of legal and regulatory requirements regarding health data	3 (4.7)
Data privacy and security measures	2 (3.1)

Patterns in structural challenges varied according to the stakeholder role. Similar to the trends observed in individual skills and expertise, data users and health data access bodies tended to express greater confidence in recognizing and addressing data quality issues, likely reflecting their more technical and analytical orientations. In contrast, data holders reported a broader spectrum of structural challenges and skill gaps, consistent with the wider range of responsibilities encompassed within this group.

### Organizational Support and Tools

**Figure 2:**
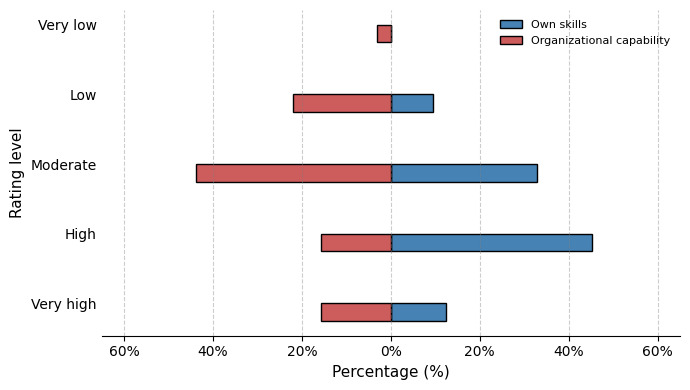
Organizational readiness for managing health data quality, including perceived data quality literacy, clarity of roles, and presence of dedicated governance structures.

While some organizations perceived themselves as having strong data quality literacy, many rated themselves lowly. Approximately a quarter of respondents rated their organization’s literacy as low or very low. Furthermore, only 15.6% (10/64; 95% CI 8.7%-26.6%) reported that the roles and responsibilities for data quality were clearly defined. In total, the organizations that 68.8% (44/64; 95% CI 56.6%-78.8%) of respondents were affiliated with lacked a dedicated data quality manager or data quality team, and most respondents described governance structures as vague or inconsistent. This ambiguity appeared to limit the effectiveness of data quality efforts (Figure 2).

A wide variety of tools were reported, from scripting and analytics tools such as Power BI and R to frameworks such as the principles of findability, accessibility, interoperability, and reusability and the ISO 8000 standard, but only a small majority (7/64, 10.9%; 95% CI 5.3%-21.1%) found them very effective. Most said that they were only moderately useful, suggesting a gap between tool availability and practical utility. Key organizational challenges included incomplete datasets, lack of system-wide consistency, insufficient staff training, and outdated infrastructure.

Stakeholder differences also emerged: health data access bodies generally viewed their organizations more positively, whereas data holders and users more often pointed to governance gaps. Overall, the findings highlight a need for better-defined roles, coordinated efforts within the infrastructures, and more strategic support for data quality at the organizational level.

### Learning Needs and Preferences

In the survey, the respondents expressed a clear preference for hands-on, flexible learning formats. In-person workshops and tutorials were the most favored, followed by webinars and self-paced courses. Reading materials were the least preferred. A total of 71.9% (46/64; 95% CI 59.9%-81.4%) of respondents said that they could dedicate 1 to 2 hours per week to training, with none willing to exceed 5 hours, highlighting the need for time-efficient formats.

When choosing a learning program, participants prioritized schedule flexibility, ongoing support, and user-friendly training platforms. Access to tutors, training affordability, and certification also mattered, although less strongly.

## Discussion

### Principal Findings

This study examined skill gaps, training needs, and organizational readiness for managing health data quality among European health data stakeholders with a focus on the 3 EHDS-defined roles: data holders, data users, and health data access bodies. Three principal findings emerged. First, despite frequent engagement with health data and widespread prior experience with data quality tasks, many stakeholders identified skill gaps, particularly in applying data quality metrics, auditing and reporting, and metadata management, suggesting that practical exposure does not automatically translate into systematic competence. Second, organizational readiness was generally low. Most organizations lacked clearly defined data quality roles or dedicated personnel, pointing to a structural deficit that individual training alone cannot solve. Third, data holders consistently reported greater difficulty in resolving data quality issues than data users or health data access bodies, a pattern with direct implications for where capacity-building investment should be prioritized under the EHDS.

As health data are being increasingly used for research, policymaking, clinical validation, and innovation, there is growing attention on the quality of those data [7]. Health data stakeholders increasingly find themselves working with datasets that were generated in contexts far removed from their own, often without full understanding of and transparency into how data were collected, managed, or curated in earlier stages of the data life cycle [20]. This lack of data provenance introduces ambiguity and risk, limiting the ability to fully trust or reuse the data effectively [9].

The survey results revealed that individuals often felt confident in addressing data quality issues: most had hands-on experience in identifying and solving problems, and many rated their own knowledge as moderate to high. However, this perceived capability tends to apply primarily to technical problems that can be easily assessed and influenced, such as fixing inconsistent data formats, identifying missing values, or cleaning duplicate entries. These issues, while important, often represent only the surface of deeper data quality concerns rooted in processes in the context of data capture, metadata, or management and governance practices.

Persistent challenges, as described by many respondents, arise from a lack of data provenance, information on a dataset’s origin, context, applied data standards, or operational workflows. Without provenance information and life cycle transparency, data users may misinterpret or mishandle data quality issues, and their ability to resolve underlying issues is constrained [21]. Embedding a provenance chain into organizational processes can improve life cycle transparency, enable more accurate quality assessments, and ensure that datasets are fit for their intended reuse [22]. While many individuals viewed themselves as moderately skilled, they simultaneously identified broader skill gaps within and across organizations, suggesting that stakeholders may be technically literate but lack the systemic, provenance-based insight needed to address the root causes of data quality issues.

A closer look at differences across the 3 EHDS-defined stakeholder types reveals that data holders, typically responsible for initial data capture and curation, reported more unresolved quality issues and expressed greater difficulty in addressing them. This contrasts with health data access bodies and data users, who may be further along the data life cycle and positioned to apply stronger analytical capabilities. However, this analytical strength does not always compensate for poor upstream quality or missing contextual information [21,23,24]. These findings underscore the importance of targeted education for data holders specifically. Equipping them with a stronger understanding of how data collection practices influence downstream use could help mitigate avoidable quality issues early in the life cycle before they propagate to users and access bodies. Although direct comparisons with other data quality educational studies are limited, the importance of a life cycle–based perspective is consistently emphasized in the literature [21,23,24]. Several studies highlight the lack of transparency in data quality assessment and reporting, which often reflects a limited understanding of the data’s origin, context, and transformation over time, reinforcing the need to embed life cycle thinking into both technical practices and capacity-building efforts [20,22]. Mapping training recommendations directly to the top gaps identified in this survey, priority investment should focus on (1) applied training in data quality metrics and audit frameworks, which together represent the 2 most frequently cited skill deficits; (2) metadata management and documentation practices, reflecting the third most cited gap and a foundational requirement for the EHDS quality labeling process under Article 78 [11]; and (3) governance and role definition training targeted specifically at data holders, who showed the greatest organizational unpreparedness. These priorities should inform the design of educational programs under the QUANTUM project and beyond.

### Limitations

Several limitations should be acknowledged. First, the convenience sampling approach via QUANTUM-affiliated networks likely resulted in selection bias toward individuals who are already more engaged with and knowledgeable about health data quality. Therefore, true gaps in the broader European health data workforce may be larger than observed. Second, the relatively small sample (N=64) and uneven country distribution, with Croatia, Belgium, and Italy overrepresented, limit the generalizability of the findings and preclude robust inferential conclusions. The uneven distribution reflects the geographic concentration of consortium partners rather than the true distribution of EHDS stakeholders. Third, all outcomes relied on self-reported perceptions of skills and organizational readiness. Self-assessment is subject to several types of bias: respondents may systematically over- or underestimate their capabilities [25]. Future studies should incorporate objective skill assessments or triangulate self-reports with organizational records. Fourth, item-level missing data were present at a very low rate. The Little MCAR test suggested a missing at random pattern; however, multiple imputation confirmed that complete-case estimates were robust. Fifth, the descriptive-only analytical approach means that observed differences between stakeholder groups cannot be statistically validated. Future studies with larger probability samples should apply inferential methods to test whether these patterns hold more broadly. Sixth, the survey instrument was purpose built and not formally validated.

### Conclusions

This study provides, to our knowledge, one of the first empirical characterizations of data quality skill gaps and training needs specifically within the 3 EHDS-defined stakeholder groups. Unlike prior work addressing data quality challenges in generic or single-stakeholder contexts, this study maps capacity deficits to the specific roles and regulatory obligations defined under the EHDS legislative framework, enabling differentiated, role-specific responses. The findings make 3 key contributions to the field. First, they demonstrate that practical experience with health data does not guarantee systematic data quality competence, a distinction with implications for how capacity-building programs should be designed. Second, they reveal that organizational barriers, including the absence of clearly defined roles and dedicated governance structures, represent a structural deficit that cannot be resolved through individual training alone and requires institutional investment. Third, the study provides a prioritized training agenda mapped directly to the most prevalent skill deficits, offering a practical evidence base for stakeholders working under the EHDS. In practical terms, these findings call for the immediate development of role-specific, time-efficient training programs for data holders, as well as policy-level attention to data quality governance structures within organizations participating in the EHDS. Ensuring that capacity-building efforts are embedded in EHDS implementation rather than treated as a secondary concern will be essential to making the Article 78 data quality labeling framework a tool for genuine improvement rather than a compliance formality.

## Appendix

Multimedia Appendix 1 Respondents’ self-reported ability to resolve data quality issues effectively.

## Abbreviations

EHDS: European Health Data Space
i~HD: European Institute for Innovation Through Health Data
MCAR: missing completely at random
QUANTUM: Quality, Utility, and Maturity Measured; Developing a Data Quality and Utility Label for the European Health Data Space
