# Supplementary figures and images for: Health Data Quality Skill Gaps and Training Needs Among European Health Data Stakeholders: Cross-Sectional Survey
Source: J Med Internet Res. 2026 Jul 6;28:e86878. doi: 10.2196/86878 (PMC13386117; doi:10.2196/86878)

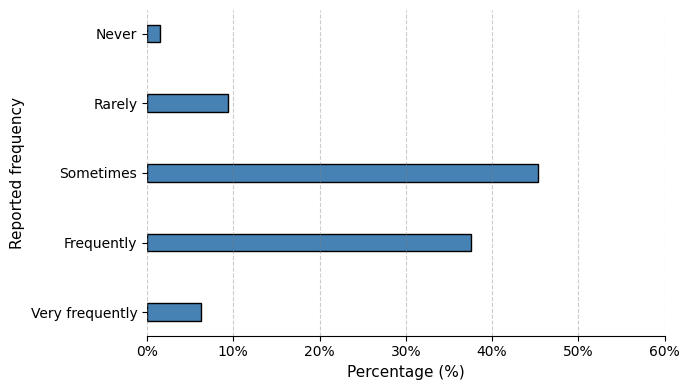

Supplement: Multimedia Appendix 1 [file jmir_v28i1e86878_app1.png]
